# Arborvitae (*Thuja plicata*) essential oil significantly inhibited critical inflammation- and tissue remodeling-related proteins and genes in human dermal fibroblasts

**DOI:** 10.1016/j.biopen.2017.02.003

**Published:** 2017-02-20

**Authors:** Xuesheng Han, Tory L. Parker

**Affiliations:** dōTERRA International, LLC, 389 S. 1300 W., Pleasant Grove, UT 84062, USA

**Keywords:** Arborvitae essential oil, Vascular cell adhesion molecule 1, Intracellular cell adhesion molecule 1, Interferon gamma-induced protein 10, Interferon-inducible T-cell chemoattractant, Collagen-III, AEO, Arborvitae (*Thuja plicata*) essential oil

## Abstract

Arborvitae (*Thuja plicata*) essential oil (AEO) is becoming increasingly popular in skincare, although its biological activity in human skin cells has not been investigated. Therefore, we sought to study AEO's effect on 17 important protein biomarkers that are closely related to inflammation and tissue remodeling by using a pre-inflamed human dermal fibroblast culture model. AEO significantly inhibited the expression of vascular cell adhesion molecule 1 (VCAM-1), intracellular cell adhesion molecule 1 (ICAM-1), interferon gamma-induced protein 10 (IP-10), interferon-inducible T-cell chemoattractant (I-TAC), monokine induced by interferon gamma (MIG), and macrophage colony-stimulating factor (M-CSF). It also showed significant antiproliferative activity and robustly inhibited collagen-I, collagen-III, plasminogen activator inhibitor-1 (PAI-1), and tissue inhibitor of metalloproteinase 1 and 2 (TIMP-1 and TIMP-2). The inhibitory effect of AEO on increased production of these protein biomarkers suggests it has anti-inflammatory property. We then studied the effect of AEO on the genome-wide expression of 21,224 genes in the same cell culture. AEO significantly and diversely modulated global gene expression. Ingenuity pathway analysis (IPA) showed that AEO robustly affected numerous critical genes and signaling pathways closely involved in inflammatory and tissue remodeling processes. The findings of this study provide the first evidence of the biological activity and beneficial action of AEO in human skin cells.

## Highlights

• Arborvitae essential oil (AEO) had significant antiproliferative activity.
• AEO inhibited VCAM-1, ICAM-1, IP-10, I-TAC, MIG, and M-CSF.
• AEO inhibited col-I and -III, PAI-1, and TIMP-1 and -2.
• AEO diversely modulated genome-wide gene expression.

## 1 Introduction

Arborvitae (*Thuja plicata*), also known as western red cedar, and its essential oils have been traditionally used as a natural insect repellent and wood preservative, primarily because of its insecticidal and antimicrobial property [1], [2], [3]. Recently, the topical use of Arborvitae essential oil (AEO) for skincare has gained popularity. However, a literature search revealed no existing studies of the biological activities of AEO in human cells. Therefore, we evaluated the biological activities of a commercially available AEO in a pre-inflamed human dermal fibroblast culture model, which was designed to model the disease biology of chronic skin inflammation. First, we analyzed the effect of AEO on 17 important protein biomarkers that are closely related to inflammation and tissue remodeling. Then, we studied its effect on genome-wide gene expression in the same cell culture.

## 2 Materials and methods

All experiments were conducted using a BioMAP system HDF3CGF, which was designed to model the pathology of chronic inflammation in a robust and reproducible manner. The system comprises three components: a cell type, stimuli to create the disease environment, and a set of biomarker (protein) readouts to examine how the treatments affected the disease environment [4].

### 2.1 Cell culture

Primary human neonatal fibroblasts (HDFs) were prepared as previously described [5] and were plated under low serum conditions for 24 h before stimulation with a mixture of interleukin (IL)-1β, tumor necrosis factor (TNF)-α, interferon (IFN)-ϒ, basic fibroblast growth factor (bFGF), epidermal growth factor (EGF), and platelet-derived growth factor (PDGF). The cell culture and stimulation conditions for the HDF3CGF assays have been described in detail elsewhere and were performed in a 96-well plate [5], [6].

### 2.2 Protein-based readouts

Direct enzyme-linked immunosorbent assay (ELISA) was used to measure the biomarker levels of cell-associated and cell membrane targets. Soluble factors in the supernatants were quantified using either homogeneous time-resolved fluorescence (HTRF) detection, bead-based multiplex immunoassay, or capture ELISA. The adverse effects of the test agents on cell proliferation and viability (cytotoxicity) were measured using the sulforhodamine B (SRB) assay. For proliferation assays, the cells were cultured and measured after 72 h, which is optimal for the HDF3CGF system, and the detailed procedure has been described in a previous study [5]. Measurements were performed in triplicate wells, and a glossary of the biomarkers used in this study is provided in Supplementary Table S1.

### 2.3 RNA isolation

Total RNA was isolated from cell lysates using the Zymo *Quick-RNA* MiniPrep kit (Zymo Research Corp., Irvine, CA, USA) according to the manufacturer's instructions. RNA concentration was determined using a NanoDrop ND-2000 system (Thermo Fisher Scientific). RNA quality was assessed using a Bioanalyzer 2100 (Agilent Technologies, Santa Clara, CA, USA) and an Agilent RNA 6000 Nano kit. All samples had an A260/A280 ratio between 1.9 and 2.1 and a RIN score >8.0.

### 2.4 Microarray analysis of genome-wide gene expression

The effect of 0.011% AEO on the expression of 21,224 genes was evaluated in the HDF3CGF system after a 24-h treatment. Samples for microarray analysis were processed by Asuragen, Inc. (Austin, TX, USA) according to the company's standard operating procedures. Biotin-labeled cRNA was prepared from 200 ng of total RNA using an Illumina TotalPrep RNA Amplification kit (Thermo Fisher Scientific) and one round of amplification. The cRNA yields were quantified using ultraviolet (UV) spectrophotometry, and the distribution of the transcript sizes was assessed using the Agilent Bioanalyzer 2100. Labeled cRNA (750 ng) was used to probe Illumina human HT-12 v4 expression bead chips (Illumina, Inc., San Diego, CA, USA). Hybridization, washing, staining with streptavidin-conjugated cyanine-3, and scanning of the Illumina arrays were carried out according to the manufacturer's instructions. The Illumina BeadScan software was used to produce the data files for each array; the raw data were extracted using Illumina BeadStudio software.

The raw data were uploaded into R [6] and analyzed for quality-control metrics using the beadarray package [7]. The data were normalized using quantile normalization [8], and then re-annotated and filtered to remove probes that were non-specific or mapped to intronic or intragenic regions [9]. The remaining probe sets comprised the data set for the remainder of the analysis. The fold-change expression for each set was calculated as the log_2_ ratio of AEO to the vehicle control. These fold-change values were uploaded onto Ingenuity Pathway Analysis (IPA, QIAGEN, Redwood City, CA, USA, www.qiagen.com/ingenuity) to generate the networks and pathway analyses.

### 2.5 Reagents

AEO (dōTERRA Intl., UT, USA) was diluted in dimethyl sulfoxide (DMSO) to 8× the specified concentrations (final DMSO concentration in culture media was no more than 0.1% [v/v]). Then, 25 μL of each 8× solution was added to the cell culture to obtain a final volume of 200 μL, and DMSO (0.1%) served as the vehicle control. The gas chromatography-mass spectrometry (GC–MS) analysis of AEO indicated that it mainly contained methyl thujate (53%) and smaller amounts of numerous other aromatic molecules.

## 3 Results and discussion

### 3.1 Bioactivity profile of AEO in pre-inflamed HDFs

**Fig. 1:**
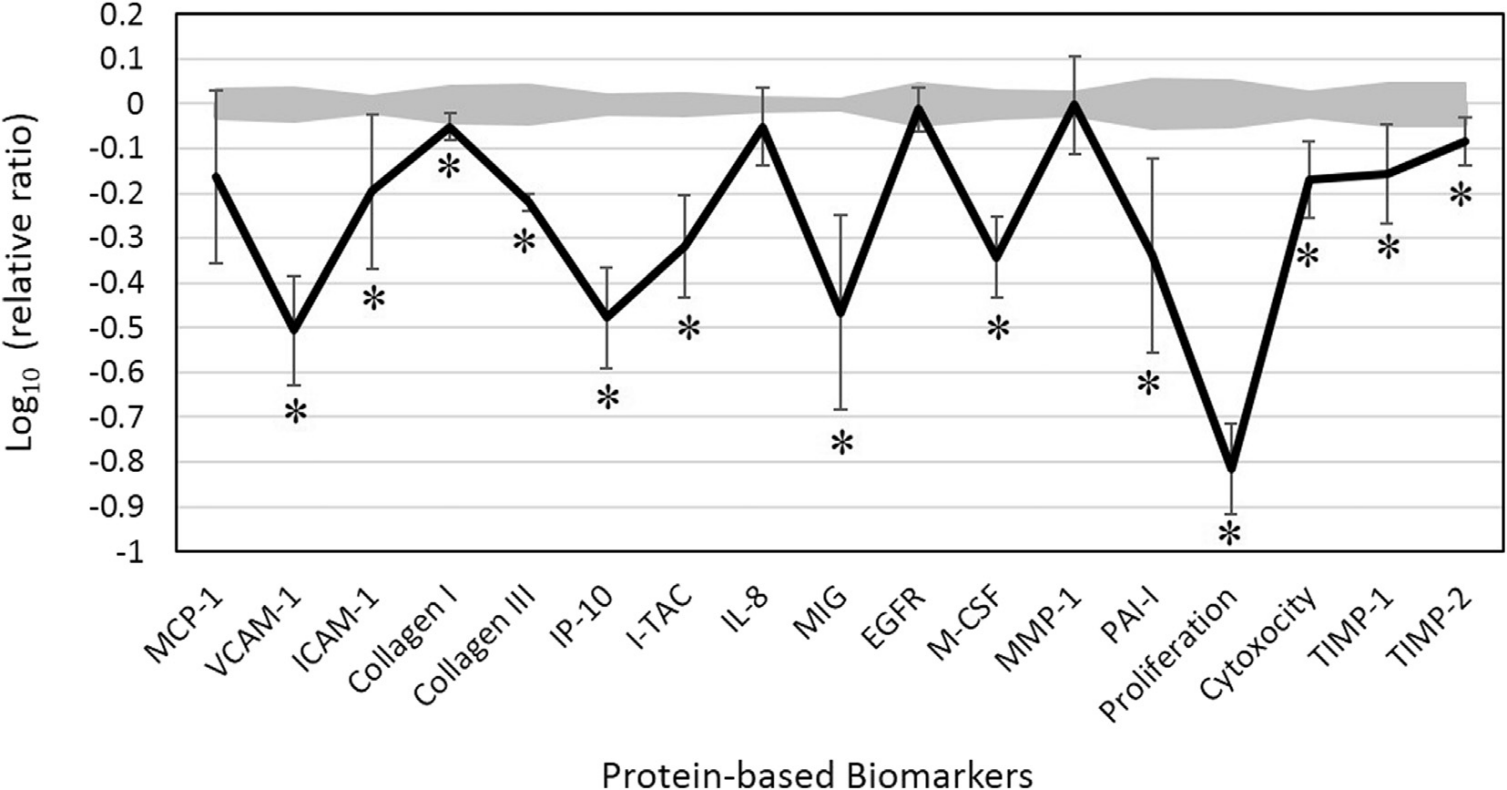
**The bioactivity profile of Arborvitae (*Thuja plicata*) essential oil (AEO, 0.011%, v/v in dimethyl sulfoxide, DMSO) in human dermal fibroblast culture (HDF3CGF).** X-axis denotes protein-based biomarker readouts. Y-axis denotes the relative expression levels of biomarkers compared with those of vehicle controls, in log form. Vehicle control values are shaded gray, with 95% significance envelope. * indicates a biomarker designated with “key activity”: biomarker value was significantly different (p < 0.05) from that of vehicle controls at the studied concentration, outside of the significance envelope, with an effect size of at least 10% (>0.05 log ratio units). MCP-1, monocyte chemoattractant protein; VCAM-1, vascular cell adhesion molecule 1; ICAM-1, intracellular cell adhesion molecule 1; IP-10, interferon gamma-induced protein 10; I-TAC, interferon-inducible T-cell alpha chemoattractant; IL-8, interleukin-8; MIG, monokine induced by gamma interferon; EGFR, epidermal growth factor; M-CSF, macrophage colony-stimulating factor; MMP-1, matrix metalloproteinase 1; PAI-1, plasminogen activator inhibitor 1; TIMP, tissue inhibitor of metalloproteinase.

We analyzed the biological activity of AEO by using an HDF3CGF cell system, which simulated the microenvironment of inflamed human skin cells with already boosted immune responses and inflammatory levels. None of the four studied concentrations (0.011, 0.0037, 0.0012, and 0.00041%, v/v) was overtly cytotoxic, and therefore, the activity of 0.011% concentration was included for analysis. Key activities of biomarkers were designated if biomarker values were significantly different (p < 0.05) from those of vehicle controls, outside of the significance envelope, with an effect size of at least 10% (>0.05 log ratio units, Fig. 1) and are discussed below.

The expressions of several inflammatory biomarkers, such as vascular cell adhesion molecule 1 (VCAM-1), intracellular cell adhesion molecule 1 (ICAM-1), interferon gamma-induced protein 10 (IP-10), interferon-inducible T-cell chemoattractant (I-TAC), and monokine induced by interferon gamma (MIG), significantly decreased in response to AEO (Fig. 1). Specifically, the levels of these protein biomarkers were already highly elevated in the pre-stimulated inflamed dermal fibroblasts. The inhibitory effects of AEO on the increased production of proinflammatory biomarkers suggest that it might possess anti-inflammatory properties.

AEO also showed significant antiproliferative activity in dermal fibroblasts, as measured using the SRB proliferation assay 72 h after treatment. The levels of five tissue remodeling molecules—collagen-I, collagen-III, plasminogen activator inhibitor-1 (PAI-1), and tissue inhibitor of metalloproteinase 1 and 2 (TIMP-1 and TIMP-2)—significantly decreased in response to AEO treatment. AEO also significantly inhibited the level of macrophage colony-stimulating factor (M-CSF), a cytokine that mediates macrophage differentiation and thus, immunomodulation. It is noteworthy that the inhibitory effects of AEO on the increased production of these protein biomarkers were concentration-dependent. AEO inhibited all these factors, which suggests that it might play important roles in tissue remodeling and immunomodulation, and thus, the wound healing processes. These effects of AEO are presumably mediated by slowing down the tissue repair process, which reduces the chance of scar formation or improper chronic wound healing [10], [11].

Recent studies on the essential oils of *T. plicata-*related species, their major active components, or both have shown preliminary evidence of their therapeutic efficacy and safety in disease models [12], [13], [14]. We conducted a literature search and found that no study has been conducted on the effects of AEO or its major component methyl thujate in human cells or similar models. Therefore, to the best of our knowledge, the current study is the first evidence of the biological activities of AEO in a human skin disease model, which suggests their anti-inflammatory, immunomodulatory, and tissue-remodeling properties in the human skin.

### 3.2 Effects of AEO on genome-wide gene expression

We then analyzed the effect of 0.011% AEO (the highest studied non-cytotoxic concentration in these cells) on the RNA expression of 21,224 genes in the same cells. The results showed the significantly diverse regulatory effect of AEO on human genes, with numerous genes being either upregulated or downregulated. Among the 200 most-regulated genes (with a fold-change ratio of expression over the vehicle control of ≥|1.5|) by AEO, the majority (121 out of 200 genes) were significantly downregulated (Table S2). A cross-comparison of the protein and gene expression data revealed that AEO significantly inhibited both the protein and gene expression levels of *VCAM-1*, *IP-10*, and *I-TAC*. This suggests that AEO might play a profound role in regulating these three important players.

**Fig. 2:**
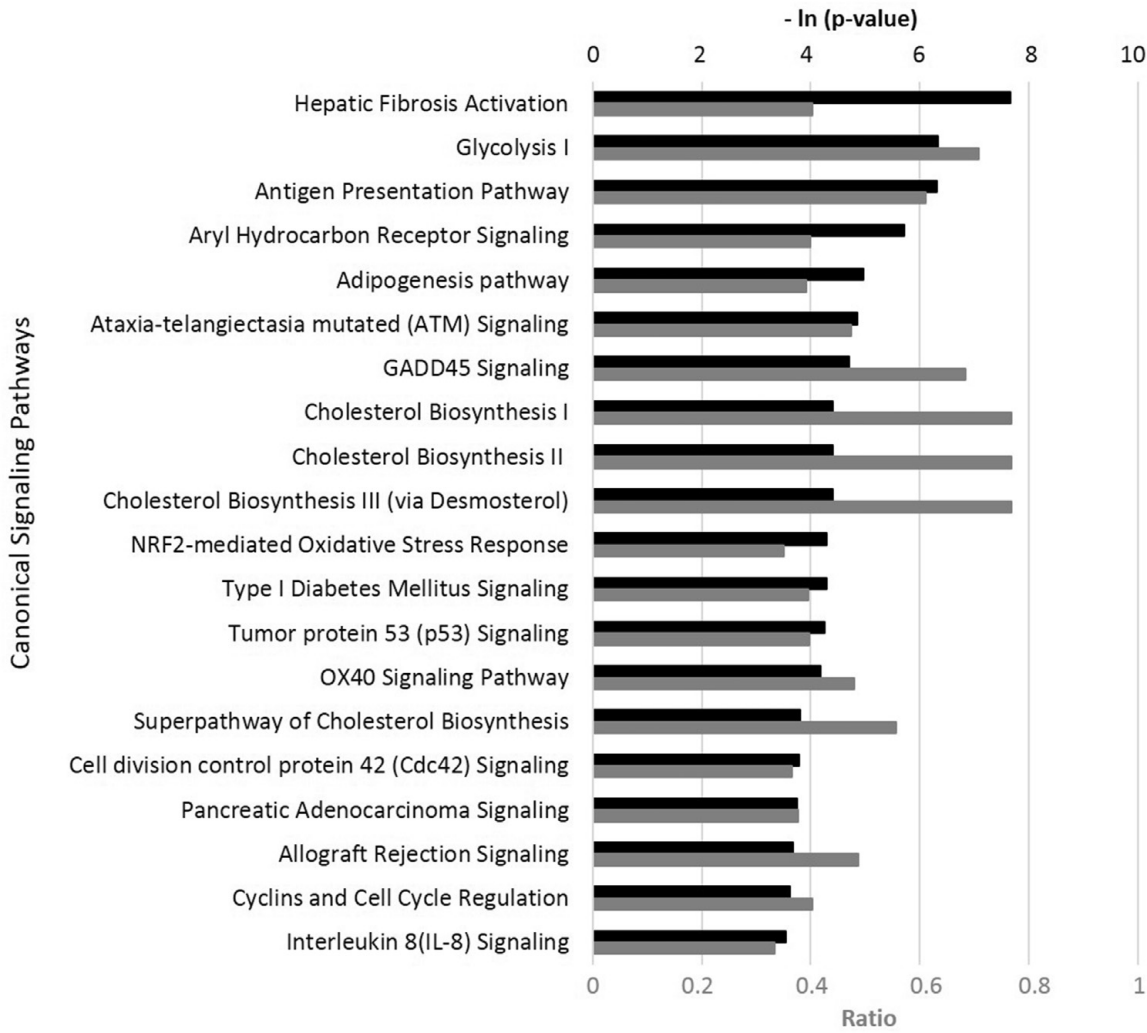
**Top 20 canonical pathways matching bioactivity profile of Arborvitae (*Thuja plicata*) essential oil (AEO) in gene expression in HDF3CGF system produced using Ingenuity Pathway Analysis (IPA, QIAGEN,**www.qiagen.com/ingenuity**).** The p-value was calculated using right-tailed Fisher's exact test. The p-value measures how likely the observed association between a specific pathway and the dataset would be if it were only due to random chance. The smaller the p-value (the bigger – ln [p-value], indicated by black bars) of the pathway, the more significantly it matches the bioactivity of AEO. Ratio, indicated using the gray bar, was calculated by dividing the number of genes from AEO dataset that participate in a canonical pathway by the total number of genes in that pathway. GADD45, growth arrest and DNA damage-inducible 45; OX40, tumor necrosis factor receptor superfamily, member 4.

IPA showed that the bioactivity of AEO significantly overlapped with numerous canonical pathways from the literature-validated database analysis (Fig. 2). Many of these signaling pathways are closely related to inflammation, immunomodulation, and tissue remodeling. Overall, AEO appeared to inhibit these signaling pathways in the highly inflamed human skin cells, suggesting it has potential anti-inflammatory and immunomodulatory effects (see Supplementary Materials for more information).

## 4 Conclusions

To the best of our knowledge, this study provides the first evidence of the biological activities of AEO in highly inflamed human skin cells. The findings show that AEO significantly inhibited numerous protein and genes involved in inflammation, immune responses, and tissue remodeling. In addition, AEO diversely and significantly modulated global gene expression. Furthermore, AEO robustly affected various important signaling pathways in human cells. These findings provide the first evidence for the therapeutic potential of AEO in human skin cell inflammation. Further studies on the mechanism of action and clinical efficacy of AEO are required before drawing definite conclusions about its therapeutic properties.

## Conflict of interest

X.H. and T.P. are employees of dōTERRA, where the study agent AEO was manufactured.

## References

[1] Guleria S., Kumar A., Tiku A.K. (2008). Chemical composition and fungitoxic activity of essential oil of *Thuja orientalis* L. grown in the North-Western Himalaya. Z. Naturforsch. C. J. Biosci..

[2] Tsiri D., Graikou K., Pobłocka-Olech L., Krauze-Baranowska M., Spyropoulos C., Chinou I. (2009). Chemosystematic value of the essential oil composition of *Thuja* species cultivated in Poland-antimicrobial activity. Molecules.

[3] Hudson J., Kuo M., Vimalanathan S. (2011). The antimicrobial properties of cedar leaf (*Thuja plicata*) oil; a safe and efficient decontamination agent for buildings. Int. J. Environ. Res. Public Health.

[4] Berg E.L., Yang J., Melrose J., Nguyen D., Privat S., Rosler E., Kunkel E.J., Ekins S. (2010). Chemical target and pathway toxicity mechanisms defined in primary human cell systems. J. Pharmacol. Toxicol. Methods.

[5] Bergamini G., Bell K., Shimamura S., Werner T., Cansfield A., Müller K., Perrin K.,J., Rau C., Ellard K., Hopf C., Doce C., Leggate D., Mangano R., Mathieson T., O'Mahony A., Plavec I., Rharbaoui F., Reinhard F., Savitski M.M., Ramsden N., Hirsch E., Drewes G., Rausch O., Bantscheff M., Neubauer G. (2012). A selective inhibitor reveals PI3Kγ dependence of T(H)17 cell differentiation. Nat. Chem. Biol..

[6] R Development Core Team (2011). R: a Language and Environment for Statistical Computing.

[7] Dunning M.J., Smith M.L., Ritchie M.E., Tavaré S. (2007). beadarray: R classes and methods for Illumina bead-based data. Bioinforma. Oxf. Engl..

[8] Bolstad B.M., Irizarry R.A., Astrand M., Speed T.P. (2003). A comparison of normalization methods for high-density oligonucleotide array data based on variance and bias. Bioinforma. Oxf. Engl..

[9] Barbosa-Morais N.L., Dunning M.J., Samarajiwa S.A., Darot J.F.J., Ritchie M.E., Lynch A.G., Tavaré S. (2010). A re-annotation pipeline for Illumina BeadArrays: improving the interpretation of gene expression data,. Nucl. Acids Res..

[10] Diegelmann R.F., Evans M.C. (2004). Wound healing: an overview of acute, fibrotic and delayed healing. Front. Biosci..

[11] Eming S.A., Krieg T., Davidson J.M. (2007). Inflammation in wound repair: molecular and cellular mechanisms. J. Invest. Dermatol.

[12] Naser B., Bodinet C., Tegtmeier M., Lindequist U. (2005). *Thuja occidentalis* (Arborvitae): a review of its pharmaceutical, pharmacological and clinical properties. Evid. Based Complement. Altern. Med..

[13] Kim K.H., Moon E., Kim S.Y., Choi S.U., Son M.W., Choi E.Z., Lee K.R. (2013). Bioactive sesquiterpenes from the essential oil of *Thuja orientalis*. Planta Med..

[14] Küpeli Akkol E., İlhan M., Ayşe Demirel M., Keleş H., Tümen I., Süntar (2015). *Thuja occidentalis* L. and its active compound, α-thujone: promising effects in the treatment of polycystic ovary syndrome without inducing osteoporosis. J. Ethnopharmacol..

