# Supplementary material for: Arborvitae (Thuja plicata) essential oil significantly inhibited critical inflammation- and tissue remodeling-related proteins and genes in human dermal fibroblasts
Source: Biochim Open. 2017 Feb 20;4:56–60. doi: 10.1016/j.biopen.2017.02.003 (PMC5801906; doi:10.1016/j.biopen.2017.02.003)

Table S1. Glossary of biomarkers of system HDF3CGF used in the study

| **Readout** | **Description** |
| --- | --- |
| **CCL2/MCP-1** | MCP-1 system is a chemokine that mediates recruitment of monocytes and T cells into sites of inflammation. MCP-1 is categorized as an inflammation-related activity in the HDF3CGF system modeling Th1 inflammation involved in wound healing and matrix remodeling. |
| **CD106/VCAM-1** | VCAM-1 is a cell adhesion molecule that mediates adhesion of monocytes and T cells to endothelial cells. VCAM-1 is categorized as an inflammation-related activity. |
| **CD54/ICAM-1** | ICAM-1 is a cell adhesion molecule that mediates leukocyte-endothelial cell adhesion and leukocyte recruitment. ICAM-1 is categorized as an inflammation-related activity. |
| **Collagen I** | Collagen I is involved in tissue remodeling and fibrosis, and is the most common fibrillar collagen that is found in skin, bone, tendons and other connective tissues. Collagen I is categorized as a tissue remodeling-related activity. |
| **Collagen III** | Collagen III is an extracellular matrix protein and fibrillar collagen found in extensible connective tissues (skin, lung and vascular system) and is involved in cell adhesion, cell migration, tissue remodeling. Collagen III is categorized as a tissue remodeling-related activity. |
| **CXCL10/IP-10** | IP-10 is a chemokine that mediates T cell, monocyte and dendritic cell chemotaxis. IP-10 is categorized as an inflammation-related activity. |
| **CXCL11/I-TAC** | I-TAC is a chemokine that mediates T cell and monocyte chemotaxis. I-TAC is categorized as an inflammation-related activity. |
| **CXCL8/IL-8** | IL-8 is a chemokine that mediates neutrophil recruitment into acute inflammatory sites. IL-8 is categorized as an inflammation-related activity. |
| **CXCL9/MIG** | MIG is a chemokine that mediates T cell recruitment. MIG is categorized as an inflammation-related activity. |
| **EGFR** | EGFR is a cell surface receptor for epidermal growth factor involved in cell proliferation during development as well as tumor growth. EGFR is involved in Epithelial cell proliferation, epithelial cell differentiation keratinocyte proliferation, tissue remodeling. EGFR is categorized as a tissue remodeling-related activity. |
| **M-CSF** | M-CSF is a secreted and cell surface cytokine that mediates macrophage differentiation. M-CSF is categorized as an immune modulation-related activity. |
| **MMP-1** | MMP-1 is an interstitial collagenase that degrades collagens I, II and III and is involved in the process of tissue remodeling. MMP-1 is categorized as a tissue remodeling-related activity. |
| **PAI-I** | PAI-I is a serine proteinase inhibitor and inhibitor of tissue plasminogen activator (tPA) and urokinase (uPA) and is involved in tissue remodeling and fibrinolysis. PAI-I is categorized as a tissue remodeling-related activity. |
| **Proliferation_72hr** | Proliferation_72hr in the HDF3CGF system is a measure of dermal fibroblast proliferation which is important to the process of wound healing and fibrosis. |
| **SRB** | SRB is a measure of the total protein content of dermal fibroblasts. Cell viability of adherent cells is measured by Sulforhodamine B (SRB) staining, a method that determines cell density by measuring total protein content of test wells. |
| **TIMP-1** | TIMP-1 is a tissue inhibitor of matrix metalloprotease-7 (MMP-7) and other MMPs, and is involved in tissue remodeling, angiogenesis and fibrosis. TIMP-1 is categorized as a tissue remodeling-related activity. |
| **TIMP-2** | TIMP-2 is a tissue inhibitor of matrix metalloproteases and is involved in tissue remodeling, angiogenesis and fibrosis. TIMP-2 is categorized as a tissue remodeling-related activity. |

Table S2. Top 200 genes impacted by AEO

| **Illumina Gene ID** | **Fold Change in Log_2_ form** | **Gene Definition** |
| --- | --- | --- |
| HSPA6 | 59.62 | Homo sapiens heat shock 70kDa protein 6 (HSP70B') (HSPA6), mRNA. |
| HS.545589 | 47.02 | Human small nuclear RNA U6atac, partial sequence |
| RNU6ATAC | 41.10 | Homo sapiens RNA, U6atac small nuclear (U12-dependent splicing) (RNU6ATAC), small nuclear RNA. |
| RMRP | 34.91 | Homo sapiens RNA component of mitochondrial RNA processing endoribonuclease (RMRP), RNase MRP RNA. |
| FOS | 34.21 | Homo sapiens v-fos FBJ murine osteosarcoma viral oncogene homolog (FOS), mRNA. |
| RGS2 | 30.83 | Homo sapiens regulator of G-protein signalling 2, 24kDa (RGS2), mRNA. |
| ZCCHC12 | 22.87 | Homo sapiens zinc finger, CCHC domain containing 12 (ZCCHC12), mRNA. |
| RPPH1 | 19.76 | Homo sapiens ribonuclease P RNA component H1 (RPPH1), RNase P RNA. |
| ID2 | 18.60 | Homo sapiens inhibitor of DNA binding 2, dominant negative helix-loop-helix protein (ID2), mRNA. |
| ID2 | 16.45 | Homo sapiens inhibitor of DNA binding 2, dominant negative helix-loop-helix protein (ID2), mRNA. |
| GADD45B | 15.64 | Homo sapiens growth arrest and DNA-damage-inducible, beta (GADD45B), mRNA. |
| ARC | 14.99 | Homo sapiens activity-regulated cytoskeleton-associated protein (ARC), mRNA. |
| HSPA7 | 13.03 | Homo sapiens heat shock 70kDa protein 7 (HSP70B) (HSPA7), non-coding RNA. |
| GADD45G | 11.49 | Homo sapiens growth arrest and DNA-damage-inducible, gamma (GADD45G), mRNA. |
| FOSB | 9.61 | Homo sapiens FBJ murine osteosarcoma viral oncogene homolog B (FOSB), mRNA. |
| MIR886 | 8.22 | Homo sapiens microRNA 886 (MIR886), microRNA. |
| RGS4 | 7.39 | Homo sapiens regulator of G-protein signalling 4 (RGS4), mRNA. |
| VTRNA1-1 | 7.29 | Homo sapiens vault RNA 1-1 (VTRNA1-1), non-coding RNA. |
| HSPA6 | 6.69 | Homo sapiens heat shock 70kDa protein 6 (HSP70B') (HSPA6), mRNA. |
| AKR1C4 | 6.41 | Homo sapiens aldo-keto reductase family 1, member C4 (chlordecone reductase; 3-alpha hydroxysteroid dehydrogenase, type I; dihydrodiol dehydrogenase 4) (AKR1C4), mRNA. |
| ID3 | 6.31 | Homo sapiens inhibitor of DNA binding 3, dominant negative helix-loop-helix protein (ID3), mRNA. |
| RASD1 | 6.17 | Homo sapiens RAS, dexamethasone-induced 1 (RASD1), mRNA. |
| HSPA1A | 5.89 | Homo sapiens heat shock 70kDa protein 1A (HSPA1A), mRNA. |
| MMP10 | 5.62 | Homo sapiens matrix metallopeptidase 10 (stromelysin 2) (MMP10), mRNA. |
| HSPA1B | 5.58 | Homo sapiens heat shock 70kDa protein 1B (HSPA1B), mRNA. |
| SLC30A1 | 5.52 | Homo sapiens solute carrier family 30 (zinc transporter), member 1 (SLC30A1), mRNA. |
| HMOX1 | 5.37 | Homo sapiens heme oxygenase (decycling) 1 (HMOX1), mRNA. |
| SNORA57 | 5.19 | Homo sapiens small nucleolar RNA, H/ACA box 57 (SNORA57), small nucleolar RNA. |
| TDO2 | 5.19 | Homo sapiens tryptophan 2,3-dioxygenase (TDO2), mRNA. |
| BAMBI | 5.14 | Homo sapiens BMP and activin membrane-bound inhibitor homolog (Xenopus laevis) (BAMBI), mRNA. |
| VTRNA1-2 | 4.97 | Homo sapiens vault RNA 1-2 (VTRNA1-2), non-coding RNA. |
| AKR1C2 | 4.89 | Homo sapiens aldo-keto reductase family 1, member C2 (dihydrodiol dehydrogenase 2; bile acid binding protein; 3-alpha hydroxysteroid dehydrogenase, type III) (AKR1C2), transcript variant 1, mRNA. XM_943424 XM_943425 XM_943427 |
| ALDH1A1 | 4.76 | Homo sapiens aldehyde dehydrogenase 1 family, member A1 (ALDH1A1), mRNA. |
| SLC30A1 | 4.63 | Homo sapiens solute carrier family 30 (zinc transporter), member 1 (SLC30A1), mRNA. |
| CRYAB | 4.48 | Homo sapiens crystallin, alpha B (CRYAB), mRNA. |
| ID1 | 4.36 | Homo sapiens inhibitor of DNA binding 1, dominant negative helix-loop-helix protein (ID1), transcript variant 2, mRNA. |
| SNORD104 | 4.30 | Homo sapiens small nucleolar RNA, C/D box 104 (SNORD104), small nucleolar RNA. |
| ALDH1A1 | 4.25 | Homo sapiens aldehyde dehydrogenase 1 family, member A1 (ALDH1A1), mRNA. |
| KLF4 | 4.04 | Homo sapiens Kruppel-like factor 4 (gut) (KLF4), mRNA. |
| SLC7A11 | 4.04 | Homo sapiens solute carrier family 7, (cationic amino acid transporter, y+ system) member 11 (SLC7A11), mRNA. |
| RDH10 | 3.96 | Homo sapiens retinol dehydrogenase 10 (all-trans) (RDH10), mRNA. |
| SLC2A3 | 3.94 | Homo sapiens solute carrier family 2 (facilitated glucose transporter), member 3 (SLC2A3), mRNA. |
| ALB | 3.92 | Homo sapiens albumin (ALB), mRNA. |
| HS.406790 | 3.88 | Homo sapiens mRNA full length insert cDNA clone EUROIMAGE 2005635 |
| FZD8 | 3.77 | Homo sapiens frizzled homolog 8 (Drosophila) (FZD8), mRNA. |
| HIST1H3C | 3.63 | Homo sapiens histone cluster 1, H3c (HIST1H3C), mRNA. |
| GDF15 | 3.56 | Homo sapiens growth differentiation factor 15 (GDF15), mRNA. |
| COL3A1 | 3.50 | Homo sapiens collagen, type III, alpha 1 (COL3A1), mRNA. |
| VAMP8 | 3.47 | Homo sapiens vesicle-associated membrane protein 8 (endobrevin) (VAMP8), mRNA. |
| HSPA2 | 3.47 | Homo sapiens heat shock 70kDa protein 2 (HSPA2), mRNA. |
| GADD45A | 3.43 | Homo sapiens growth arrest and DNA-damage-inducible, alpha (GADD45A), mRNA. |
| PLAU | 3.41 | Homo sapiens plasminogen activator, urokinase (PLAU), mRNA. |
| GADD45A | 3.40 | Homo sapiens growth arrest and DNA-damage-inducible, alpha (GADD45A), mRNA. |
| ADH1A | 3.37 | Homo sapiens alcohol dehydrogenase 1A (class I), alpha polypeptide (ADH1A), mRNA. |
| HSPA2 | 3.24 | Homo sapiens heat shock 70kDa protein 2 (HSPA2), mRNA. |
| SNAI1 | 3.23 | Homo sapiens snail homolog 1 (Drosophila) (SNAI1), mRNA. |
| CYP26B1 | 3.22 | Homo sapiens cytochrome P450, family 26, subfamily B, polypeptide 1 (CYP26B1), mRNA. |
| DNAJB1 | 3.20 | Homo sapiens DnaJ (Hsp40) homolog, subfamily B, member 1 (DNAJB1), mRNA. |
| IL24 | 3.20 | Homo sapiens interleukin 24 (IL24), transcript variant 2, mRNA. |
| RGS16 | 3.19 | Homo sapiens regulator of G-protein signalling 16 (RGS16), mRNA. |
| RNY4 | 3.14 | Homo sapiens RNA, Ro-associated Y4 (RNY4), small cytoplasmic RNA. |
| EGR1 | 3.13 | Homo sapiens early growth response 1 (EGR1), mRNA. |
| LOC643311 | 3.10 | PREDICTED: Homo sapiens hypothetical protein LOC643311 (LOC643311), mRNA. |
| CA2 | 3.09 | Homo sapiens carbonic anhydrase II (CA2), mRNA. |
| FAM167A | 3.04 | Homo sapiens family with sequence similarity 167, member A (FAM167A), mRNA. |
| MIR221 | 3.03 | Homo sapiens microRNA 221 (MIR221), microRNA. |
| SCG5 | 3.03 | Homo sapiens secretogranin V (7B2 protein) (SCG5), mRNA. |
| C8ORF13 | 2.94 | Homo sapiens chromosome 8 open reading frame 13 (C8orf13), mRNA. |
| RAB38 | 2.93 | Homo sapiens RAB38, member RAS oncogene family (RAB38), mRNA. |
| PDK4 | 2.89 | Homo sapiens pyruvate dehydrogenase kinase, isozyme 4 (PDK4), mRNA. |
| HIST1H2BG | 2.83 | Homo sapiens histone cluster 1, H2bg (HIST1H2BG), mRNA. |
| CXCL12 | 2.81 | Homo sapiens chemokine (C-X-C motif) ligand 12 (stromal cell-derived factor 1) (CXCL12), transcript variant 1, mRNA. |
| TSPAN13 | 2.80 | Homo sapiens tetraspanin 13 (TSPAN13), mRNA. |
| NXF1 | 2.80 | Homo sapiens nuclear RNA export factor 1 (NXF1), transcript variant 1, mRNA. |
| AGPAT9 | 2.79 | Homo sapiens 1-acylglycerol-3-phosphate O-acyltransferase 9 (AGPAT9), mRNA. |
| DHDH | 2.79 | Homo sapiens dihydrodiol dehydrogenase (dimeric) (DHDH), mRNA. |
| SNORD12C | 2.76 | Homo sapiens small nucleolar RNA, C/D box 12C (SNORD12C), small nucleolar RNA. |
| COL1A1 | 2.73 | Homo sapiens collagen, type I, alpha 1 (COL1A1), mRNA. |
| IL24 | 2.72 | Homo sapiens interleukin 24 (IL24), transcript variant 1, mRNA. |
| KIFC1 | -2.72 | Homo sapiens kinesin family member C1 (KIFC1), mRNA. |
| GCH1 | -2.72 | Homo sapiens GTP cyclohydrolase 1 (GCH1), transcript variant 4, mRNA. |
| PSTPIP2 | -2.74 | Homo sapiens proline-serine-threonine phosphatase interacting protein 2 (PSTPIP2), mRNA. |
| CYB5A | -2.74 | Homo sapiens cytochrome b5 type A (microsomal) (CYB5A), transcript variant 2, mRNA. |
| CXCL6 | -2.75 | Homo sapiens chemokine (C-X-C motif) ligand 6 (granulocyte chemotactic protein 2) (CXCL6), mRNA. |
| AKAP12 | -2.76 | Homo sapiens A kinase (PRKA) anchor protein (gravin) 12 (AKAP12), transcript variant 2, mRNA. |
| CCL11 | -2.76 | Homo sapiens chemokine (C-C motif) ligand 11 (CCL11), mRNA. |
| CFH | -2.77 | Homo sapiens complement factor H (CFH), transcript variant 2, mRNA. |
| NUSAP1 | -2.77 | Homo sapiens nucleolar and spindle associated protein 1 (NUSAP1), transcript variant 2, mRNA. |
| HECW2 | -2.77 | Homo sapiens HECT, C2 and WW domain containing E3 ubiquitin protein ligase 2 (HECW2), mRNA. |
| CEACAM1 | -2.77 | Homo sapiens carcinoembryonic antigen-related cell adhesion molecule 1 (biliary glycoprotein) (CEACAM1), transcript variant 2, mRNA. |
| ADAMDEC1 | -2.78 | Homo sapiens ADAM-like, decysin 1 (ADAMDEC1), mRNA. |
| SCARA3 | -2.79 | Homo sapiens scavenger receptor class A, member 3 (SCARA3), transcript variant 2, mRNA. |
| RRM2 | -2.79 | Homo sapiens ribonucleotide reductase M2 polypeptide (RRM2), mRNA. |
| METTL7A | -2.79 | Homo sapiens methyltransferase like 7A (METTL7A), mRNA. |
| OAS1 | -2.80 | Homo sapiens 2',5'-oligoadenylate synthetase 1, 40/46kDa (OAS1), transcript variant 3, mRNA. |
| CDCA3 | -2.81 | Homo sapiens cell division cycle associated 3 (CDCA3), mRNA. |
| RUNX3 | -2.82 | Homo sapiens runt-related transcription factor 3 (RUNX3), transcript variant 2, mRNA. |
| WNK4 | -2.84 | Homo sapiens WNK lysine deficient protein kinase 4 (WNK4), mRNA. |
| HAO2 | -2.86 | Homo sapiens hydroxyacid oxidase 2 (long chain) (HAO2), transcript variant 2, mRNA. |
| P4HA1 | -2.87 | Homo sapiens procollagen-proline, 2-oxoglutarate 4-dioxygenase (proline 4-hydroxylase), alpha polypeptide I (P4HA1), transcript variant 1, mRNA. |
| PBK | -2.87 | Homo sapiens PDZ binding kinase (PBK), mRNA. |
| HLA-DRB4 | -2.88 | Homo sapiens major histocompatibility complex, class II, DR beta 4 (HLA-DRB4), mRNA. |
| HLA-DRB6 | -2.88 | Homo sapiens major histocompatibility complex, class II, DR beta 6 (pseudogene) (HLA-DRB6), non-coding RNA. |
| KIAA0101 | -2.91 | Homo sapiens KIAA0101 (KIAA0101), transcript variant 1, mRNA. |
| FAM20A | -2.91 | Homo sapiens family with sequence similarity 20, member A (FAM20A), mRNA. |
| NDP | -2.92 | Homo sapiens Norrie disease (pseudoglioma) (NDP), mRNA. |
| C9ORF135 | -2.93 | Homo sapiens chromosome 9 open reading frame 135 (C9orf135), mRNA. |
| RNF146 | -2.94 | Homo sapiens ring finger protein 146 (RNF146), mRNA. |
| ACSL5 | -2.94 | Homo sapiens acyl-CoA synthetase long-chain family member 5 (ACSL5), transcript variant 2, mRNA. |
| TOP2A | -2.95 | Homo sapiens topoisomerase (DNA) II alpha 170kDa (TOP2A), mRNA. |
| P4HA2 | -2.95 | Homo sapiens prolyl 4-hydroxylase, alpha polypeptide II (P4HA2), transcript variant 2, mRNA. |
| KIF11 | -2.97 | Homo sapiens kinesin family member 11 (KIF11), mRNA. |
| CFH | -2.98 | Homo sapiens complement factor H (CFH), transcript variant 2, mRNA. |
| ANLN | -2.99 | Homo sapiens anillin, actin binding protein (ANLN), mRNA. |
| MXRA5 | -3.00 | Homo sapiens matrix-remodelling associated 5 (MXRA5), mRNA. |
| CXCL11 | -3.00 | Homo sapiens chemokine (C-X-C motif) ligand 11 (CXCL11), mRNA. |
| HIST1H4C | -3.01 | Homo sapiens histone cluster 1, H4c (HIST1H4C), mRNA. |
| SAA1 | -3.04 | Homo sapiens serum amyloid A1 (SAA1), transcript variant 2, mRNA. |
| MUC1 | -3.05 | Homo sapiens mucin 1, cell surface associated (MUC1), transcript variant 6, mRNA. |
| SLC2A1 | -3.07 | Homo sapiens solute carrier family 2 (facilitated glucose transporter), member 1 (SLC2A1), mRNA. |
| IL18BP | -3.08 | Homo sapiens interleukin 18 binding protein (IL18BP), transcript variant A, mRNA. |
| LEPREL1 | -3.09 | Homo sapiens leprecan-like 1 (LEPREL1), mRNA. |
| BNIP3 | -3.12 | Homo sapiens BCL2/adenovirus E1B 19kDa interacting protein 3 (BNIP3), nuclear gene encoding mitochondrial protein, mRNA. |
| ALDOC | -3.15 | Homo sapiens aldolase C, fructose-bisphosphate (ALDOC), mRNA. |
| GNA15 | -3.15 | Homo sapiens guanine nucleotide binding protein (G protein), alpha 15 (Gq class) (GNA15), mRNA. |
| NOD2 | -3.15 | Homo sapiens nucleotide-binding oligomerization domain containing 2 (NOD2), mRNA. |
| HMMR | -3.20 | Homo sapiens hyaluronan-mediated motility receptor (RHAMM) (HMMR), transcript variant 1, mRNA. |
| CEP55 | -3.20 | Homo sapiens centrosomal protein 55kDa (CEP55), mRNA. |
| CFH | -3.21 | Homo sapiens complement factor H (CFH), transcript variant 1, mRNA. |
| PRC1 | -3.22 | Homo sapiens protein regulator of cytokinesis 1 (PRC1), transcript variant 2, mRNA. |
| HNMT | -3.23 | Homo sapiens histamine N-methyltransferase (HNMT), transcript variant 2, mRNA. |
| SEPT4 | -3.24 | Homo sapiens septin 4 (SEPT4), transcript variant 3, mRNA. |
| MELK | -3.26 | Homo sapiens maternal embryonic leucine zipper kinase (MELK), mRNA. |
| IGFBP7 | -3.26 | Homo sapiens insulin-like growth factor binding protein 7 (IGFBP7), mRNA. |
| INSIG2 | -3.27 | Homo sapiens insulin induced gene 2 (INSIG2), mRNA. |
| AK3L1 | -3.29 | Homo sapiens adenylate kinase 3-like 1 (AK3L1), nuclear gene encoding mitochondrial protein, transcript variant 7, mRNA. |
| SCARA3 | -3.32 | Homo sapiens scavenger receptor class A, member 3 (SCARA3), transcript variant 1, mRNA. |
| FDPS | -3.34 | Homo sapiens farnesyl diphosphate synthase (farnesyl pyrophosphate synthetase, dimethylallyltranstransferase, geranyltranstransferase) (FDPS), mRNA. |
| IFI44L | -3.35 | Homo sapiens interferon-induced protein 44-like (IFI44L), mRNA. |
| ACSL5 | -3.37 | Homo sapiens acyl-CoA synthetase long-chain family member 5 (ACSL5), transcript variant 1, mRNA. |
| IRG1 | -3.38 | PREDICTED: Homo sapiens immunoresponsive 1 homolog (mouse) (IRG1), mRNA. |
| INDO | -3.39 | Homo sapiens indoleamine-pyrrole 2,3 dioxygenase (INDO), mRNA. |
| NCAPG | -3.39 | Homo sapiens non-SMC condensin I complex, subunit G (NCAPG), mRNA. |
| HLA-DRB1 | -3.40 | Homo sapiens major histocompatibility complex, class II, DR beta 1 (HLA-DRB1), mRNA. |
| IDO1 | -3.41 | Homo sapiens indoleamine 2,3-dioxygenase 1 (IDO1), mRNA. |
| TNFSF13B | -3.41 | Homo sapiens tumor necrosis factor (ligand) superfamily, member 13b (TNFSF13B), transcript variant 1, mRNA. |
| ALDH1A3 | -3.44 | Homo sapiens aldehyde dehydrogenase 1 family, member A3 (ALDH1A3), mRNA. |
| SLC26A4 | -3.45 | Homo sapiens solute carrier family 26, member 4 (SLC26A4), mRNA. |
| IFI27 | -3.48 | Homo sapiens interferon, alpha-inducible protein 27 (IFI27), transcript variant 2, mRNA. |
| CCL3L1 | -3.51 | Homo sapiens chemokine (C-C motif) ligand 3-like 1 (CCL3L1), mRNA. |
| UBE2C | -3.53 | Homo sapiens ubiquitin-conjugating enzyme E2C (UBE2C), transcript variant 3, mRNA. |
| A2M | -3.57 | Homo sapiens alpha-2-macroglobulin (A2M), mRNA. |
| IL4I1 | -3.57 | Homo sapiens interleukin 4 induced 1 (IL4I1), transcript variant 2, mRNA. |
| CDC2 | -3.57 | Homo sapiens cell division cycle 2, G1 to S and G2 to M (CDC2), transcript variant 1, mRNA. |
| FABP3 | -3.62 | Homo sapiens fatty acid binding protein 3, muscle and heart (mammary-derived growth inhibitor) (FABP3), mRNA. |
| UBE2C | -3.67 | Homo sapiens ubiquitin-conjugating enzyme E2C (UBE2C), transcript variant 6, mRNA. |
| SEPT4 | -3.71 | Homo sapiens septin 4 (SEPT4), transcript variant 1, mRNA. |
| ALDH1A3 | -3.76 | Homo sapiens aldehyde dehydrogenase 1 family, member A3 (ALDH1A3), mRNA. |
| DEFB4 | -3.87 | Homo sapiens defensin, beta 4 (DEFB4), mRNA. |
| HLA-DRA | -3.89 | Homo sapiens major histocompatibility complex, class II, DR alpha (HLA-DRA), mRNA. |
| AK3L1 | -3.89 | Homo sapiens adenylate kinase 3-like 1 (AK3L1), nuclear gene encoding mitochondrial protein, transcript variant 6, mRNA. |
| RHOU | -3.90 | Homo sapiens ras homolog gene family, member U (RHOU), mRNA. |
| FLJ21986 | -3.93 | Homo sapiens hypothetical protein FLJ21986 (FLJ21986), mRNA. |
| TNFSF13B | -3.93 | Homo sapiens tumor necrosis factor (ligand) superfamily, member 13b (TNFSF13B), transcript variant 1, mRNA. |
| HLA-F | -3.98 | Homo sapiens major histocompatibility complex, class I, F (HLA-F), transcript variant 1, mRNA. |
| LOC730249 | -4.12 | PREDICTED: Homo sapiens similar to Immune-responsive protein 1 (LOC730249), mRNA. |
| CCL3L1 | -4.21 | Homo sapiens chemokine (C-C motif) ligand 3-like 1 (CCL3L1), mRNA. |
| CCL20 | -4.28 | Homo sapiens chemokine (C-C motif) ligand 20 (CCL20), mRNA. |
| AK3L1 | -4.29 | Homo sapiens adenylate kinase 3-like 1 (AK3L1), nuclear gene encoding mitochondrial protein, transcript variant 7, mRNA. |
| CCL3 | -4.72 | Homo sapiens chemokine (C-C motif) ligand 3 (CCL3), mRNA. |
| HSD11B1 | -4.77 | Homo sapiens hydroxysteroid (11-beta) dehydrogenase 1 (HSD11B1), transcript variant 2, mRNA. |
| ASCL2 | -4.79 | Homo sapiens achaete-scute complex homolog 2 (Drosophila) (ASCL2), mRNA. |
| HSD11B1 | -4.87 | Homo sapiens hydroxysteroid (11-beta) dehydrogenase 1 (HSD11B1), transcript variant 2, mRNA. |
| HS.10862 | -4.89 | Homo sapiens cDNA: FLJ23313 fis, clone HEP11919 |
| LOC730415 | -4.91 | PREDICTED: Homo sapiens hypothetical LOC730415, transcript variant 2 (LOC730415), mRNA. |
| RARRES1 | -5.10 | Homo sapiens retinoic acid receptor responder (tazarotene induced) 1 (RARRES1), transcript variant 2, mRNA. |
| CX3CL1 | -5.22 | Homo sapiens chemokine (C-X3-C motif) ligand 1 (CX3CL1), mRNA. |
| CCL3L3 | -5.24 | Homo sapiens chemokine (C-C motif) ligand 3-like 3 (CCL3L3), mRNA. |
| TNFSF10 | -5.28 | Homo sapiens tumor necrosis factor (ligand) superfamily, member 10 (TNFSF10), mRNA. |
| CFB | -5.32 | Homo sapiens complement factor B (CFB), mRNA. |
| LIPG | -5.37 | Homo sapiens lipase, endothelial (LIPG), mRNA. |
| RARRES1 | -5.42 | Homo sapiens retinoic acid receptor responder (tazarotene induced) 1 (RARRES1), transcript variant 1, mRNA. |
| HSD11B1 | -5.45 | Homo sapiens hydroxysteroid (11-beta) dehydrogenase 1 (HSD11B1), transcript variant 1, mRNA. |
| PI3 | -5.52 | Homo sapiens peptidase inhibitor 3, skin-derived (SKALP) (PI3), mRNA. |
| CD38 | -5.52 | Homo sapiens CD38 molecule (CD38), mRNA. |
| SEPT4 | -5.62 | Homo sapiens septin 4 (SEPT4), transcript variant 2, mRNA. |
| ABI3BP | -5.98 | Homo sapiens ABI gene family, member 3 (NESH) binding protein (ABI3BP), mRNA. |
| CD74 | -6.12 | Homo sapiens CD74 molecule, major histocompatibility complex, class II invariant chain (CD74), transcript variant 2, mRNA. |
| SLC2A5 | -6.32 | Homo sapiens solute carrier family 2 (facilitated glucose/fructose transporter), member 5 (SLC2A5), mRNA. |
| HLA-DRA | -6.32 | Homo sapiens major histocompatibility complex, class II, DR alpha (HLA-DRA), mRNA. |
| VCAM1 | -6.34 | Homo sapiens vascular cell adhesion molecule 1 (VCAM1), transcript variant 1, mRNA. |
| MYH11 | -7.47 | Homo sapiens myosin, heavy chain 11, smooth muscle (MYH11), transcript variant SM1A, mRNA. |
| HAS3 | -7.60 | Homo sapiens hyaluronan synthase 3 (HAS3), transcript variant 1, mRNA. |
| CD74 | -7.62 | Homo sapiens CD74 molecule, major histocompatibility complex, class II invariant chain (CD74), transcript variant 1, mRNA. |
| IL32 | -7.82 | Homo sapiens interleukin 32 (IL32), transcript variant 4, mRNA. |
| UBD | -9.06 | Homo sapiens ubiquitin D (UBD), mRNA. |
| VCAM1 | -9.66 | Homo sapiens vascular cell adhesion molecule 1 (VCAM1), transcript variant 1, mRNA. |
| CXCL9 | -10.69 | Homo sapiens chemokine (C-X-C motif) ligand 9 (CXCL9), mRNA. |
| CCL5 | -24.82 | Homo sapiens chemokine (C-C motif) ligand 5 (CCL5), mRNA. |
| CCL5 | -41.09 | Homo sapiens chemokine (C-C motif) ligand 5 (CCL5), mRNA. |

Table S3. Top 20 genes regulated by AEO in the canonical Hepatic Fibrosis/Hepatic Stellate Cell Activation pathway. Fold change over vehicle was shown in log_2_ ratio form.


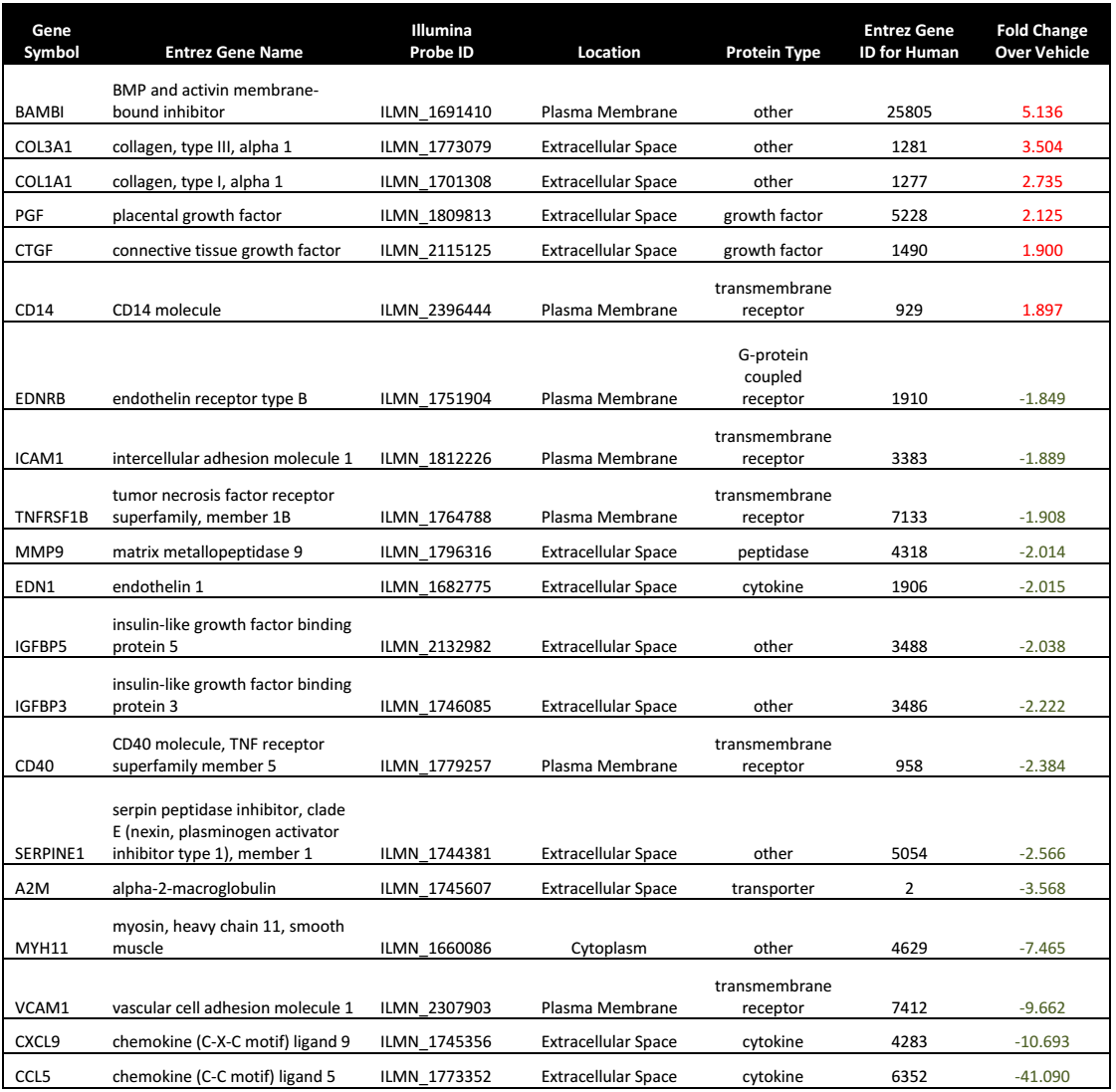


Table S4. Top 17 genes regulated by AEO in the canonical Glycolysis I pathway. Fold change over vehicle was shown in log_2_ ratio form.
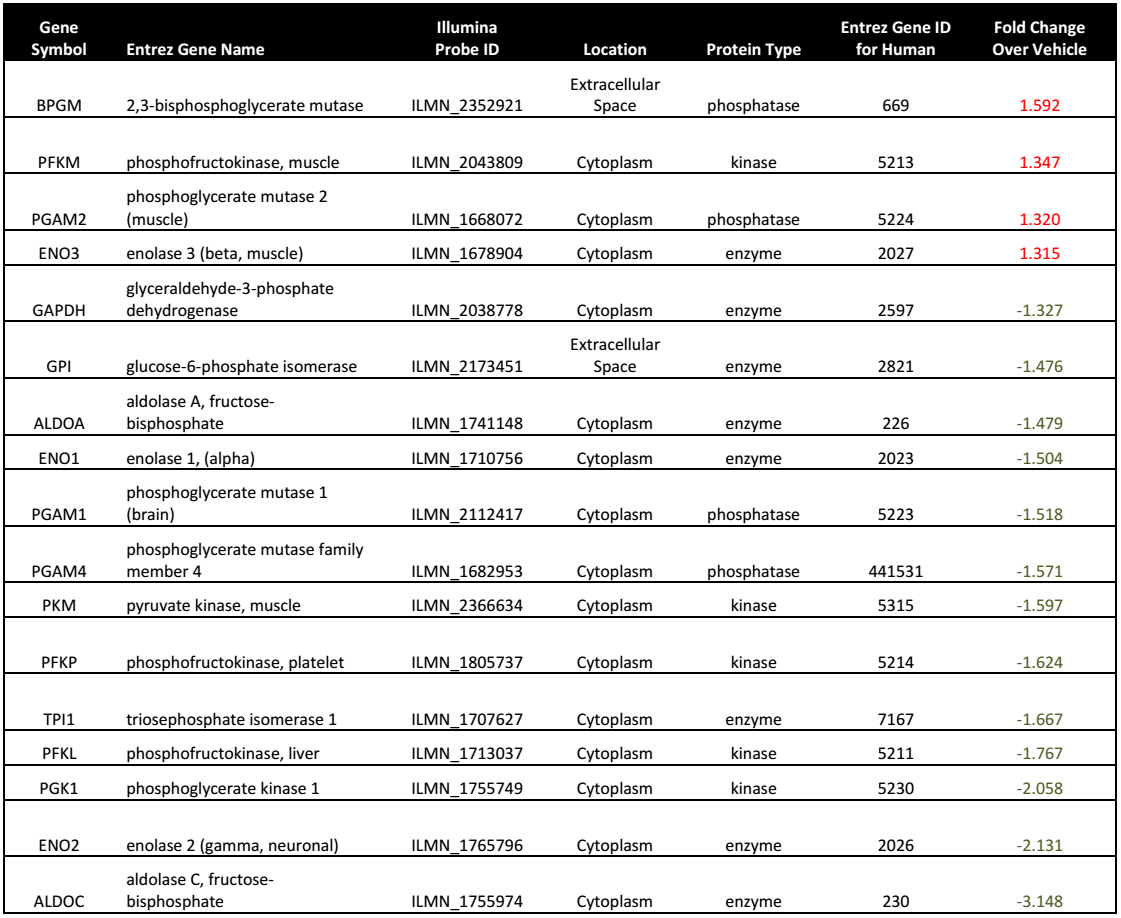


Table 5. Top 20 genes regulated by AEO in the canonical Antigen Presentation pathway. Fold change over vehicle was shown in log_2_ ratio form.


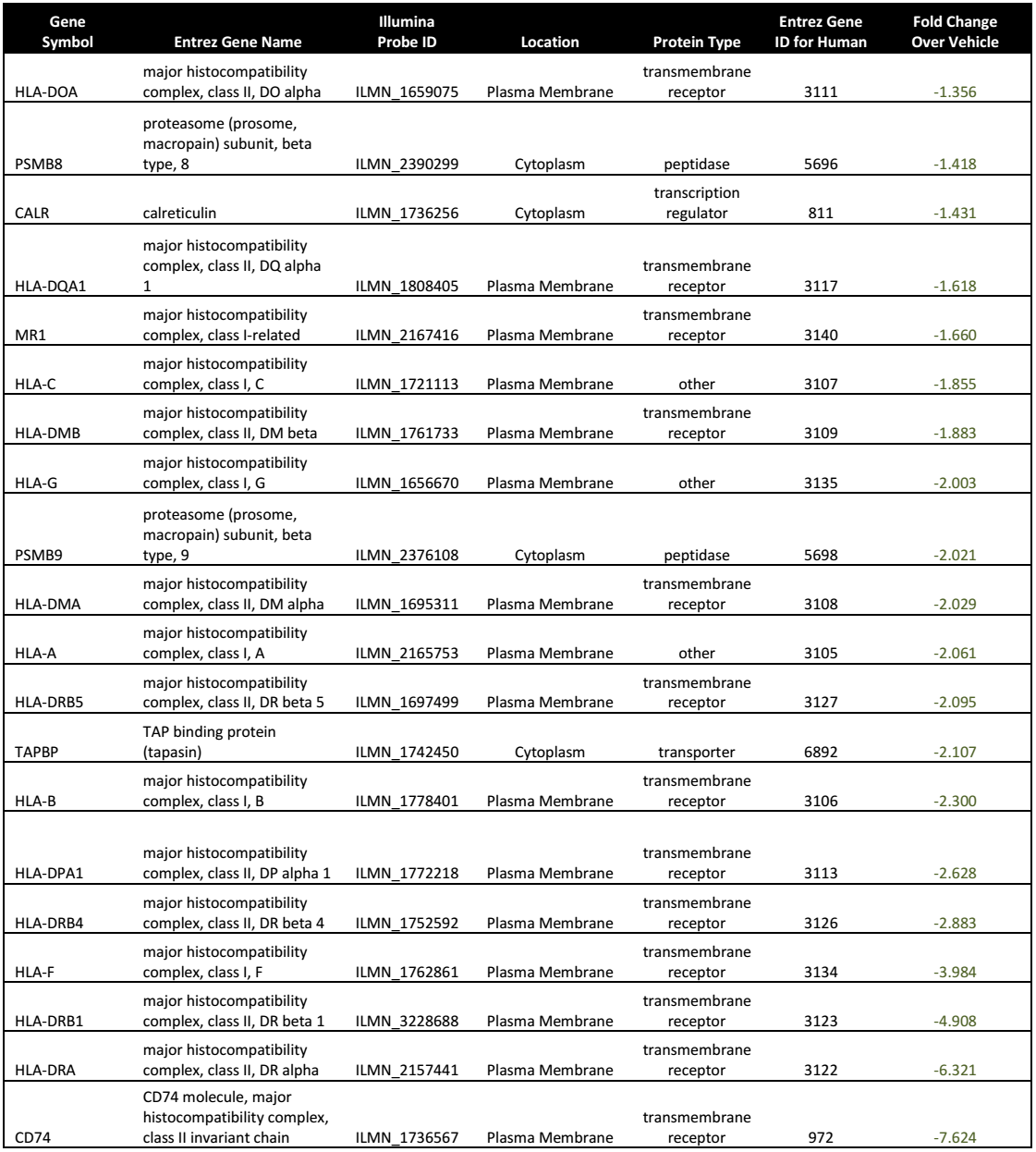


Table S6. Top 20 genes regulated by AEO in the canonical Aryl Hydrocarbon Receptor Signaling pathway. Fold change over vehicle was shown in log_2_ ratio form.


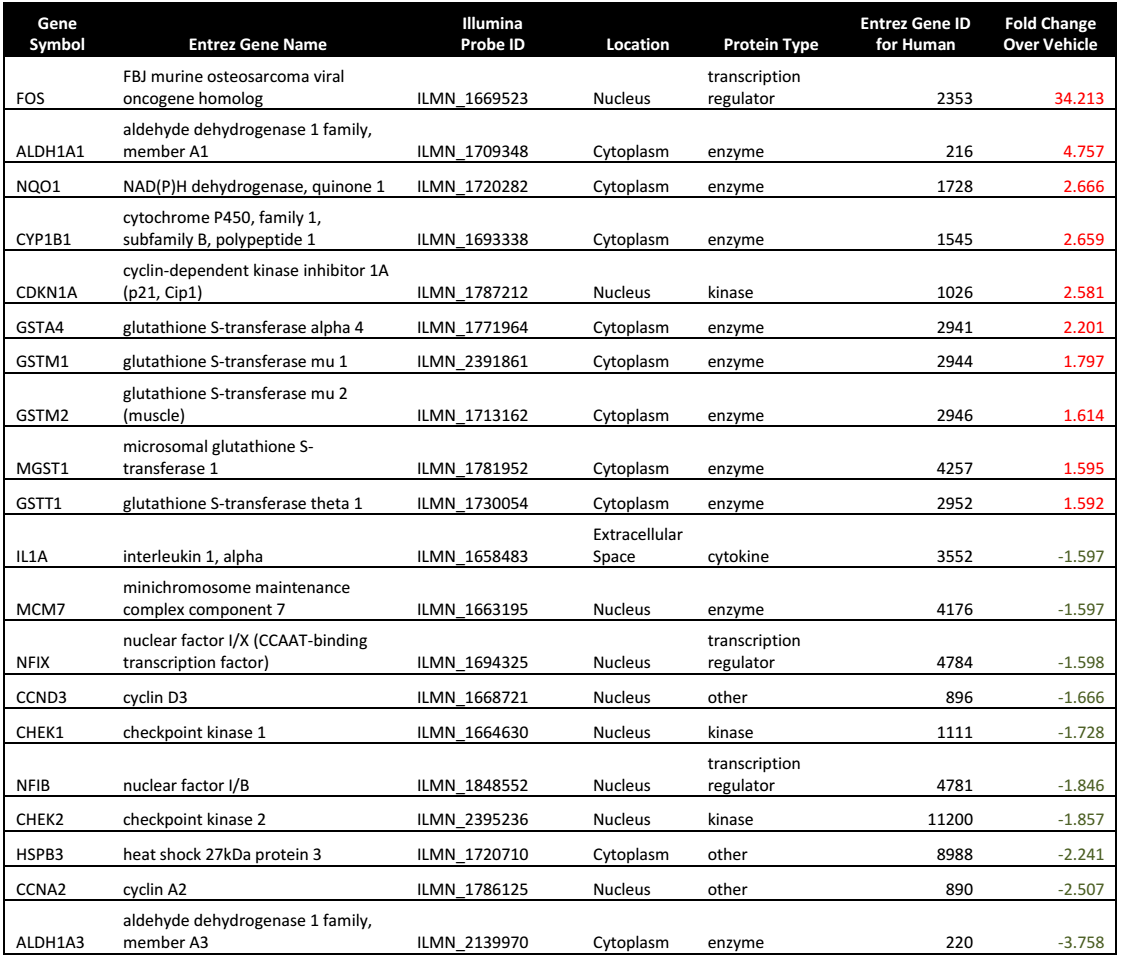

Supplement: Supplementary file 1 [file mmc1.docx]
